# Modular 3D-Printed Peg Biofilm Device for Flexible Setup of Surface-Related Biofilm Studies

**DOI:** 10.3389/fcimb.2021.802303

**Published:** 2022-02-03

**Authors:** Greta Zaborskytė, Erik Wistrand-Yuen, Karin Hjort, Dan I. Andersson, Linus Sandegren

**Affiliations:** Department of Medical Biochemistry and Microbiology, Uppsala University, Uppsala, Sweden

**Keywords:** biofilm, 3D printing, medical device, bacterial infections, silicone, *Escherichia coli*, *Klebsiella pneumoniae*

## Abstract

Medical device-related biofilms are a major cause of hospital-acquired infections, especially chronic infections. Numerous diverse models to study surface-associated biofilms have been developed; however, their usability varies. Often, a simple method is desired without sacrificing throughput and biological relevance. Here, we present an in-house developed 3D-printed device (FlexiPeg) for biofilm growth, conceptually similar to the Calgary Biofilm device but aimed at increasing ease of use and versatility. Our device is modular with the lid and pegs as separate units, enabling flexible assembly with up- or down-scaling depending on the aims of the study. It also allows easy handling of individual pegs, especially when disruption of biofilm populations is needed for downstream analysis. The pegs can be printed in, or coated with, different materials to create surfaces relevant to the study of interest. We experimentally validated the use of the device by exploring the biofilms formed by clinical strains of *Escherichia coli* and *Klebsiella pneumoniae*, commonly associated with device-related infections. The biofilms were characterized by viable cell counts, biomass staining, and scanning electron microscopy (SEM) imaging. We evaluated the effects of different additive manufacturing technologies, 3D printing resins, and coatings with, for example, silicone, to mimic a medical device surface. The biofilms formed on our custom-made pegs could be clearly distinguished based on species or strain across all performed assays, and they corresponded well with observations made in other models and clinical settings, for example, on urinary catheters. Overall, our biofilm device is a robust, easy-to-use, and relevant assay, suitable for a wide range of applications in surface-associated biofilm studies, including materials testing, screening for biofilm formation capacity, and antibiotic susceptibility testing.

## 1 Introduction

With advances in medical procedures, the use of diverse medical devices has become a new norm in temporary situations, for example, intravenous or urinary catheterization, as well as providing long-term solutions, such as implants. Unfortunately, both temporary medical devices and implants can serve as a platform for bacterial biofilm growth (Costerton et al., 2005). Biofilms can be broadly defined as microbial communities where the cells are attached to a surface or to each other and enclosed in an extracellular matrix (ECM) (Costerton et al., 1995), and they represent the predominant state of growth in the environment and during chronic infections (Hall-Stoodley et al., 2004). Device-related biofilms can reach a massive size, exceeding 1-mm thickness, due to the fact that device surfaces lack host defense mechanisms (Bjarnsholt et al., 2013). In addition, host materials, such as fibrinogen, readily coat such surfaces and facilitate bacterial attachment (Flores-Mireles et al., 2016). Established biofilms are difficult, or even impossible, to eradicate without the removal of the device due to their high tolerance to both antimicrobial treatment and immune factors (Høiby et al., 2015).

Device-related biofilms are commonly at the center of experimental biofilm studies *in vitro*, probably because the growth on artificial non-living surfaces is easier to control and analyze than, for example, growth as aggregates in sputum. Even so, biofilms and research approaches to study them are so diverse that it is impossible to have a one-size-fits-all assay, and as reviewed elsewhere, numerous published models have been extensively employed (Lebeaux et al., 2013; Guzmán-Soto et al., 2021). When choosing a model system, there are many important parameters to consider, of which biological relevance, ease of use, and high throughput are among the most important.

Some of the most commonly used high-throughput assays are the microtiter plate model (Christensen et al., 1985; O’Toole, 2011) and the Calgary biofilm device (CBD) (Ceri et al., 1999), also known by the commercial MBEC™ name, denoting its use for antibiotic susceptibility testing of biofilms (Harrison et al., 2010). While widely available, the relevance of the microtiter plate assay in biofilm studies can be questioned with regard to the surface material used (polystyrene) and the propensity of the cells to sedimentation. CBD is considered superior to the microtiter plate model, as it minimizes the attachment by sedimentation, while still retaining a high throughput 96-well format. CBD, along with the drip flow reactor, rotating disk reactor, CDC biofilm reactor, and the colony biofilm model, is approved by the American Society for Testing and Materials (ASTM) as a standard method to study the susceptibility of *Pseudomonas aeruginosa* biofilms (Gomes et al., 2018). While the microtiter plate model is mostly used in combination with crystal violet (CV) staining, the CBD adds a possibility for downstream analysis of disrupted biofilm populations, which is important when assessing the antibiotic susceptibility of biofilms.

However, there are a few practical limitations to the CBD (Azeredo et al., 2017), for instance, the lack of a convenient method for handling individual pegs. To remove a peg from the CBD lid, one has to physically break it off with pliers, thus applying mechanical force on surfaces close to the biofilm with an increased risk of unintended biofilm disruption, contamination, or loss of a peg. In addition, being a single-use device, it is rather expensive, especially if any special coatings are required. The entire device has to be sacrificed even if less than 96 biofilms are grown. To address this issue, the bead assay, also compatible with the microtiter plates, was recently developed (Konrat et al., 2016). Even though it offers flexibility in terms of up- or down-scaling, the associated biofilm processing protocol requires direct mechanical interaction with the biofilm growth surface during washing and transfer of the beads. Finally, the relevance of peg material, which in most cases is polystyrene, has also been questioned (Gomes et al., 2018).

Additive manufacturing, also known as 3D printing, serves as a fast and cost-effective way to manufacture custom-designed objects, including medically relevant products, for example, surgical guides, implants, and other devices (Paul et al., 2018). Some materials used in additive manufacturing of medical devices, such as polylactic acid polymers (Hall et al., 2021) or titanium plates (Palka et al., 2020), have recently been studied for their propensity for bacterial attachment and growth. Microfluidics systems frequently rely on 3D-printed molds for casting silicone (polydimethylsiloxane (PDMS)) chips for biofilm growth (Kim et al., 2012). Some reports on biofilm growth systems manufactured entirely by 3D printing have also started to appear. For instance, a reusable, 3D-printed flow device has been designed to study chronic wound infections (Duckworth et al., 2018), and a microfluidic flow-cell system to study dental biofilms (Kristensen et al., 2020).

To increase the versatility of common high throughput methods for surface-related biofilm growth, we designed the FlexiPeg biofilm device that can be easily manufactured by standard 3D printing. The device is composed of a lid with pegs that are inserted through a silicone mat to fit into a 96-well plate. Instead of being a single unit, the system is modular with the lid and pegs as separate units, allowing the desired number of individual pegs to be inserted at specified locations. The entire model system, including the pegs, is made out of autoclavable materials, making it reusable, thus greatly reducing the cost and the amount of plastic waste. In addition, the pegs can be printed in any desired material and with additional peg coatings, e.g., clinically relevant silicone, that are frequently used in catheters (Feneley et al., 2015). We validated the use of the FlexiPeg with different peg materials and coatings by growing biofilms of *Klebsiella pneumoniae* and *Escherichia coli*, both known to form biofilms on various medical devices (Macleod and Stickler, 2007; Wang et al., 2009; Reisner et al., 2014; Flores-Mireles et al., 2015). We demonstrate the usability of the system by viable cell counts, biomass staining, and scanning electron microscopy (SEM) imaging, and we demonstrate that this biofilm device is a robust, easy-to-use, and relevant assay adjustable to the user’s needs.

## 2 Materials and Methods

### 2.1 The Design and Manufacturing of the Model System

**Figure 1:**
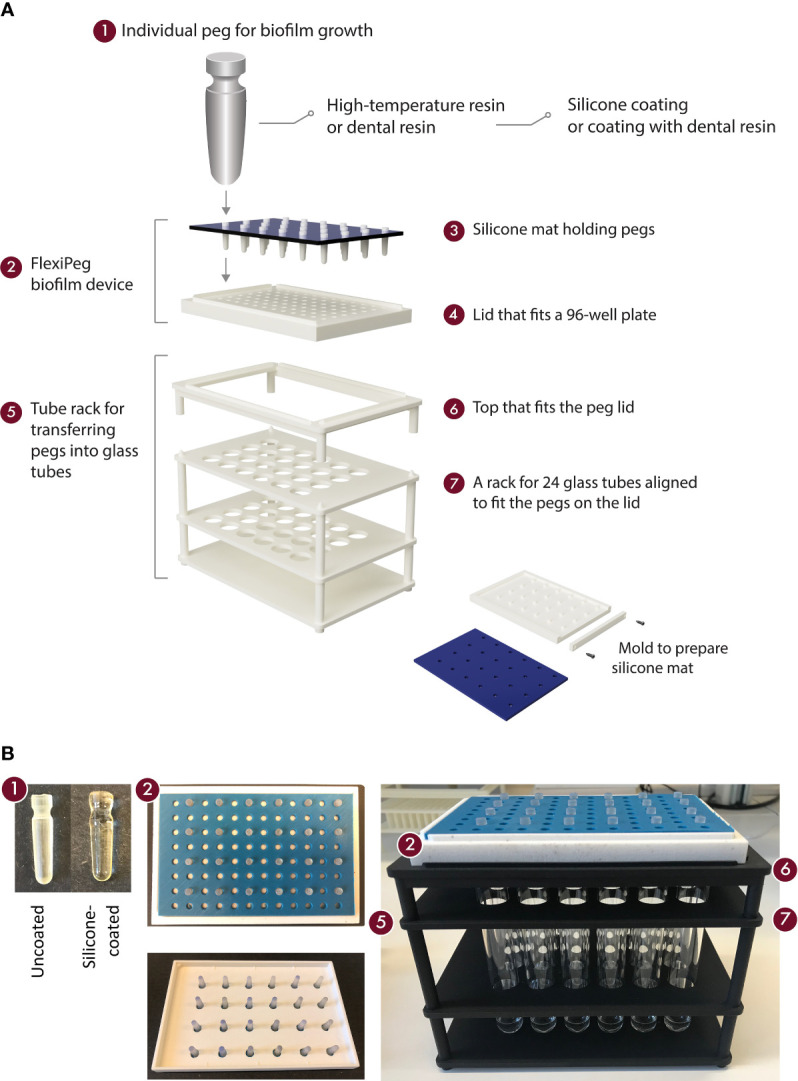
An overview of the components of the model system for biofilm growth. Schematic representation **(A)** and pictures **(B)** of the modular 3D-printed FlexiPeg device and its associated custom-made accessories. CAD drawings are available upon request.

The model system (**Figure 1**) consists of the following components: the FlexiPeg biofilm device (2) including individual pegs (1) that are inserted into a lid (4) and held in place by a silicone mat (3), as well as a rack (5) assisting the transfer of pegs into glass tubes for dispersal of biofilms. The shape and size (17.8 mm long, top diameter 4.4 mm, surface area 45 mm^2^ at 4-mm depth where most of the biofilm growth occurs) of the individual pegs were designed to match those of the CBD. The lid is available in a few variants: with 96 holes or 24 holes and with 4.6-mm-diameter holes to accommodate uncoated pegs or 5.6-mm-diameter holes for both uncoated and coated pegs. The biofilm device fits as a lid onto standard flat- or round-bottom 96-well plates (Nunc™). Pegs were printed using either high-temperature (HT) resin (High Temp, Formlabs) or biocompatible dental SG resin (Formlabs). Black resin (Formlabs) was used for printing the mold for the silicone mat. 3D printing of pegs and the mold for the silicone mat was performed at U-PRINT: Uppsala University’s 3D printing facility at the Disciplinary Domain of Medicine and Pharmacy using Formlabs Form 2 (stereolithographic technology) or Form 3 (low force stereolithography) 3D printers. 3D printing of lids and tube racks was done with polyamide (SLS) at the Materialise facility (Belgium). CAD drawings of all components are available for download here https://www.imbim.uu.se/digitalAssets/675/c_675902-l_1-k_flexipeg_cad_drawings.zip.

The silicone mat was cast in the 3D-printed mold by mixing equal amounts (10.5 ml each) of silicone base and silicone catalyst (Slöjd-Detaljer, Sweden, product number 2911-0000) and left to solidify for at least 1 h at room temperature (20°C). All parts, including pegs, peg lids, silicone mats, and tube racks, are autoclavable. After use, the pegs were washed in Contrad^®^ 70 (Decon Labs, Inc.) detergent in 2% (w/w) water solution overnight, followed by rinsing with deionized water, and if needed, 70% ethanol. Prior to use, the required pegs were assembled onto a lid with a silicone mat in place and autoclaved in a self-sealing pouch 140 × 250 mm (Eline^®^, VWR).

#### 2.1.1 Coating of Pegs With Dental Resin

The dental SG resin pegs were post-processed according to Formlabs recommendations. The printed part was dipped in liquid dental SG resin and inverted to let the resin coat the pegs under gravity for 1 h in a vacuum desiccator. The resin was then cured in a UV chamber filled with nitrogen gas for 30 min.

#### 2.1.2 Coating of Pegs With Silicone (Polydimethylsiloxane)

The peg lid (with pegs still attached to the base after 3D printing) was immersed into a mixture of silicone (PDMS, SYLGARD™ 184 silicone elastomer) and a catalyst (ratio 10:1), turned upside down, and allowed to coat the pegs by gravity at 60°C for 2 days (note: 1 day can be enough, but we found that 2 days minimized the risk of leftover sticky residue). The coating was done twice for each set of pegs. We recommend keeping the mixture of silicone and catalyst at −20°C for 1 day before the coating to make it more viscous and to achieve a more homogenous coating of the entire peg surface.

### 2.2 Bacterial Strains and Growth Conditions

We used three strains to characterize biofilm growth in the FlexiPeg biofilm device. *E. coli* CFT073 (DA47111) is a uropathogenic (UPEC) clinical strain isolated from blood and urine (Kao et al., 1997; Luo et al., 2009). *K. pneumoniae* C3091 (DA12090) is a UTI isolate originally from Walter Reed Army Medical Center (Oelschlaeger and Tall, 1997). *K. pneumoniae* strain DA69557 is a previously uncharacterized, strong biofilm-forming variant of an extended-spectrum beta-lactamase (ESBL)-producing clone from a clinical outbreak at Uppsala University Hospital (Lytsy et al., 2008; Sandegren et al., 2012). Two of the strains have been previously used in biofilm studies. The mechanisms of biofilm formation of *E. coli* CFT073 (Allsopp et al., 2010; Reisner et al., 2014; Luterbach et al., 2018) and the effects of antibiotics (Rivardo et al., 2011; Gandee et al., 2015) have been studied extensively in various biofilm models. *K. pneumoniae* C3091 (DA12090) has been used for elucidating the role of type 1 and type 3 fimbriae (Struve et al., 2003; Struve et al., 2009; Stahlhut et al., 2012) that are involved in *K. pneumoniae* biofilm formation on urinary catheters (Murphy et al., 2013) and other surfaces.

Bacterial cultures were grown in Brain Heart Infusion (BHI) broth (Oxoid), Lysogeny Broth (LB; Sigma-Aldrich), or M9 medium supplemented with 0.2% of glucose prior to and during biofilm growth. Overnight starting cultures (O/N) were grown at 37°C with 180 rpm shaking. Biological replicates in biofilm experiments denote biofilms started from independent liquid cultures that were inoculated from separate colonies.

### 2.3 General Biofilm Growth and Disruption

O/N liquid cultures were grown in BHI broth prior to inoculation of the 96-well plate. Over night cultures were diluted 100-fold or 10,000-fold and 150ul were transferred to a flat-bottom transparent 96-well plate (Nunc™), and the autoclaved lid with pegs was inserted into the wells. To avoid excess evaporation from the wells during incubation, the plate with the peg lid was put in a plastic box (optional: placing wet paper towels underneath the plate) and placed in a 37°C incubator for static incubation. If the biofilms are to be harvested afterward, we recommend placing a microtiter plate lid over the device to prevent contamination of the peg top. The peg lid was transferred to a new 96-well plate with fresh BHI broth every 24 h. At the end of incubation, the pegs were washed three times by transferring the peg lid to a 96-well plate containing 180 μl of sterile phosphate-buffered saline (PBS). Then the pegs were transferred to individual sterile glass tubes (15–16 mm diameter) by placing the peg lid on the customized tube rack and pushing the pegs into the tubes. The glass tubes need to have at least 600 µl of liquid (usually PBS, but can be a growth medium if the harvested population is going to be used for downstream experiments requiring growth). Tubes with pegs were vortexed (Vortex-Genie 2T, 230V) for 1–2 min full speed to disperse the biofilms. CFU counts were performed by preparing serial dilutions in PBS and plating appropriate dilutions on LB Agar (LA, Sigma-Aldrich) plates.

#### 2.3.1 Biofilm Disruption by Sonication

Sonication (60-kHz frequency) in a VWR ultrasonic bath was performed for 10, 20, or 30 min with pegs transferred into glass tubes (15–16 mm diameter) containing 600 µl of PBS or 0.05% Tween-20 (Sigma-Aldrich) solution. For additional disruption of biofilms, 10× trypsin (Sigma-Aldrich) treatment was done for 30 min at 37°C before sonication.

#### 2.3.2 Repeatability Testing

To assess day-to-day variation, *K. pneumoniae* DA69557 and *E. coli* CFT073 were statically grown for 48 h on HT pegs in BHI with transfer to a new medium after 24 h. The experiments were run independently on 4 days, with four (*K. pneumoniae*) or three (*E. coli*) biological replicates in each experiment.

#### 2.3.3 Influence of Peg Location

To determine whether peg location (row and column coordinates in a microtiter plate) affects biofilm growth, 24 pegs were inoculated from a single overnight culture of *K. pneumoniae* DA69557 and *E. coli* CFT073. Biofilms were allowed to form for 48 h and harvested as described in the general protocol.

### 2.4 Quantification of Biofilm Biomass by Crystal Violet Staining

Biofilms formed on pegs were washed three times by transferring the peg lid to a 96-well plate containing 180 µl of PBS. After drying the pegs for 30 min at 37°C, 180 μl of 0.1% CV (Sigma) stain was added. After 20 min of incubation at room temperature (20°C), the pegs were extensively washed (3–4×) with 180 μl of water or PBS to remove any non-bound CV stain. Then the pegs were dried for 20 min, and 200 μl of 95% ethanol (*E. coli*) or 10% acetic acid (*K. pneumoniae*) was added to solubilize the stain. The amount of bound CV was quantified by measuring absorbance at 540 nm with a Multiskan™ FC Microplate Photometer (Thermo Scientific). The average value from blank (peg incubated in growth medium for the same duration as the samples, CV-stained and stain solubilized) was subtracted from the sample values.

### 2.5 Biofilm Growth and Maturation Dynamics on the Pegs

To determine the growth and biofilm maturation on the HT and silicone-coated pegs, viable cell count (CFU per peg) and CV staining of biofilms were done at certain timepoints. CFU per peg was performed at 2, 4, 6, (8), 12, 24, 48, 72, and (96 h) (8 h for silicone-coated pegs and 96 h for both HT and silicone-coated pegs are timepoints only used for *K. pneumoniae* strains). CV staining was done at 24, 48, 72, and 96 h (*K. pneumoniae*) with an additional 12-h (HT and silicone pegs) and 36-h (silicone only) timepoints for DA69557.

### 2.6 Scanning Electron Microscopy

Biofilms were grown on the HT and silicone-coated pegs in BHI for 6, 16 (*K. pneumoniae* strains only), or 48 h with a medium change after 24 h. After washing 3× in sterile PBS, biofilms on pegs were fixed in 200 µl of 2.5% glutaraldehyde and 1% paraformaldehyde in PIPES or phosphate buffer for 1.5 h at room temperature (20°C). Then biofilms were washed 3× in 200 µl of 0.1 M of phosphate buffer (10-min incubation each time) and dehydrated by transferring the pegs to increasing concentrations of ethanol (30%, 50%, 70%, 80%, 90%, and 99.5%) with 10-min incubation at each concentration. The final incubation in 99.9% ethanol was continued for 20 min. The samples were air-dried and kept in a box with desiccator beads (Sigma Aldrich) until analysis (maximum 1 day).

The AuPd coating was carried out with a Polaron SC7640 High Resolution Sputter Coater with a plasma current of 20 mA at an applied voltage of 2 kV for 40 s, resulting in coatings of approximately 5- to 6-nm thickness. SEM images were acquired with a Zeiss Merlin field-emission SEM (FESEM) using an acceleration voltage of 2 or 3 kV and a working distance at approximately 6 mm. Sputter coating and SEM imaging were done at the MyFab facility at Ångström laboratory, Uppsala University. Control pegs without any biofilm growth were used for SEM imaging of the surface. For pegs with biofilm growth, the entire peg surface (one side which was coated with AuPd) was examined, and images were taken at approximately 4-mm distance from the tip, where most of the biofilm growth occurs.

### 2.7 Statistical Analysis

All statistical analyses were done using GraphPad Prism version 9.2.0 for macOS (GraphPad Software, San Diego, CA, USA). The performed tests are indicated for each experiment.

## 3 Results

### 3.1 Initial Validation of the FlexiPeg Device With High-Temperature Resin Pegs

**Figure 2:**
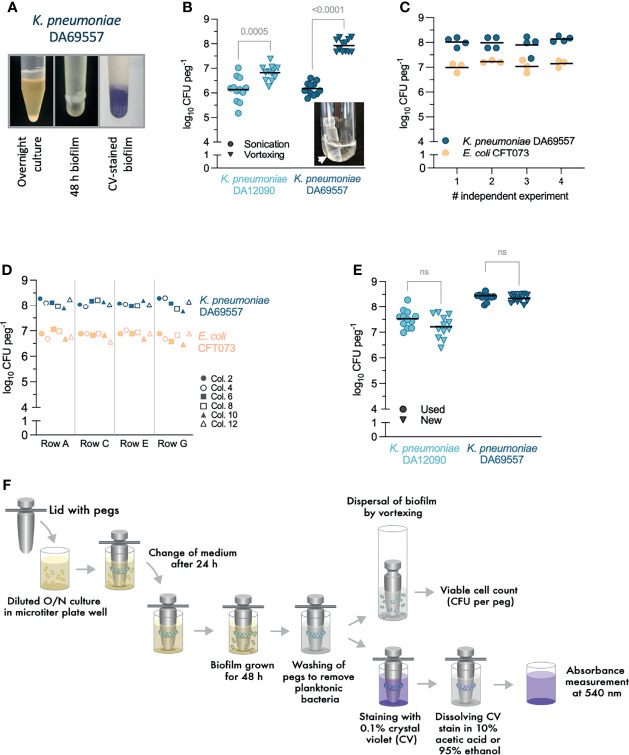
Testing and optimization of experimental protocols with high-temperature (HT) resin pegs. **(A)** *Klebsiella pneumoniae* DA69557 biofilm phenotype in a stationary liquid culture (left), biomass present on the peg after 48-h growth (middle), and biomass stained with crystal violet (CV) (right). **(B)** Comparison between vortexing (2 min) and low-frequency (60 kHz) sonication (10 min) for disruption of 48 h *K pneumoniae* biofilms from the pegs. The inset picture shows biomass on the peg visible even after a prolonged (up to 30 min) sonication. Results from three independent experiments are shown with a total of 12 biological replicates, and the lines represent the mean. Statistical significance was assessed by unpaired two-tailed Student’s t-test; difference is considered significant at p < 0.05. **(C)** Repeatability of biofilm growth and disruption by vortexing after 48 h in Brain Heart Infusion (BHI). Dots represent biological replicates with the lines indicating the mean. Statistical significance for each strain was assessed by one-way ANOVA; difference is considered statistically significant at p < 0.05. **(D)** Positional repeatability of biofilm growth in the 24-peg lid. Row letters and column (col.) numbers refer to the labeling on a microtiter plate. The result for each peg location is the mean from two independent experiments. Statistical significance was assessed by two-way ANOVA. For *K pneumoniae* DA69557, p (row factor) = 0.9027, p (column factor) = 0.3741. For *Escherichia coli* CFT073, p (row factor) = 0.2311, p (column factor) = 0.1819. Difference significant at p < 0.05. ns, denotes not significant difference. **(E)** Biofilm growth is not affected by the repeated use and washing–autoclaving cycles for pegs. The comparison was done between used (undergone at least 30 biofilm growth-washing–autoclaving cycles) and freshly printed pegs. Results from three independent experiments are shown with a total of 12 biological replicates and the mean as a line. Statistical significance assessed by paired two-tailed Student’s t-test; difference significant at p < 0.05. **(F)** General experimental workflow for biofilm growth, disruption, viable count, and staining with CV.

Both *E. coli* and *K. pneumoniae* are well known to form surface-attached biofilms, for example, on medical devices like urinary catheters (Frank et al., 2009; Reisner et al., 2014; Flores-Mireles et al., 2015). Therefore, to validate and characterize biofilm growth in the FlexiPeg biofilm device, we chose the well-studied UPEC biofilm former *E. coli* (CFT073) and two *K. pneumoniae* strains C3091 (DA12090) and DA69557. Strain DA69557 served as an indicator strain due to its strong biofilm phenotype (**Figure 2A**, left). Initial validation was done using pegs made out of HT resin that is composed of acrylic-based polymers and able to withstand autoclaving. A high amount of biomass, visible to the naked eye as well as after staining with CV, accumulated on HT pegs inoculated with DA69557 (**Figure 2A**). To avoid uneven disruption of biofilm from the pegs when harvesting biofilms, we transferred the pegs into individual glass tubes instead of another microtiter plate. To assist the transfer, we designed a custom tube rack so that the peg lid fits on top of the rack and aligns each peg to an individual tube, after which pegs are simply pushed into the tubes using sterile tweezers or a pipette tip.

Low-frequency sonication is often used to disrupt biofilms before viable cell counts and is suggested for the MBEC™ assay (Harrison et al., 2010). However, in accordance with some warnings issued by others (Azeredo et al., 2017), we observed that sonication was not efficient at disrupting biofilm produced by *K. pneumoniae* on CBD pegs or our custom 3D-printed HT pegs. This was especially evident for the strong biofilm former DA69557 with a 2-log difference in CFU per peg between sonication and vortexing and remaining biomass visible on the peg after sonication (**Figure 2B**). Increasing sonication time up to 30 min, adding Tween-20 during sonication, or treating with trypsin before sonication was still less efficient than vortexing for 2 min at full speed (**Supplementary Figure 1**). CFU counts on agar plates were consistent with cell number analysis by flow cytometry of biofilm populations disrupted by vortexing (data not shown). Collectively, these results confirmed vortexing as a more reliable technique to remove and disrupt the biofilm populations.

Biofilm growth on HT pegs, assessed by CFU per peg after vortexing, was consistent in independent experiments performed on different days (**Figure 2C**), and the location of the peg in the plate lid did not affect biofilm growth (**Figure 2D**). In addition, we did not observe any functional deterioration of HT pegs after up to 30 cycles of repeated use–washing–autoclaving (**Figure 2E**) proving that pegs are reusable, thereby greatly reducing the cost of each experiment. Although we have not experienced any variation in biofilm formation on different batches of pegs from our printing facilities, we recommend testing every new batch for consistency against old pegs. Biofilm growth was dependent on growth medium (**Supplementary Figure 2**), reflecting the responsiveness of our model to different growth conditions, which can affect both the growth of cells and production of ECM (Stoodley et al., 1998). Since rich BHI broth supported the largest population size and biofilm maturation, it was chosen as a medium for the following experiments for all strains.

After initial optimization, the general protocol for the growth and processing of biofilms was designed (**Figure 2F**). Like CBD, the FlexiPeg device is a batch-type model that accommodates a change of growth medium at any desired timepoint simply by transferring the whole peg lid with attached biofilms into a new microtiter plate with a fresh medium. This facilitates rapid washing, treatment, or change of growth medium. When disruption of biofilms is required, the pegs are transferred into the glass tubes arranged in the custom tube rack and vortexed to disrupt the biofilm. For staining with CV, the pegs can remain inserted in the lid for easier handling of multiple pegs; however, staining of single pegs is also possible. The CV stain can be dissolved by acetic acid or ethanol for biomass quantification (discussed more in later sections). Used pegs can be washed in a detergent that does not leave residue and autoclaved for new experiments.

### 3.2 Comparison of Biofilm Growth on Different Types of Pegs

**Figure 3:**
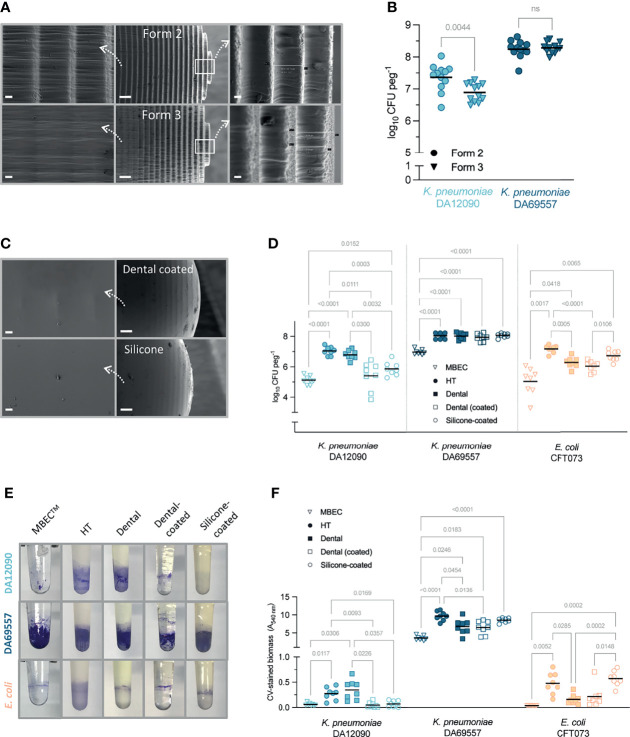
The effect of peg surface finish and material on *Klebsiella pneumoniae* and *Escherichia coli* biofilm yield. **(A)** Scanning electron microscopy (SEM) images of high-temperature (HT) resin pegs printed on Form 2 (top) and Form 3 (bottom) printer using stereolithography or low force stereolithography technology, respectively. Middle images show overall peg appearance at ×150 magnification, right side (×1,000), and the tip of the peg, left (×1,000), the surface where most of the biofilm growth occurs (approx. 4 mm from the tip). Scale bars: 20 µm (right and left panels) and 200 µm (middle). **(B)** Viable cell count for 48-h biofilms grown on HT resin pegs printed on different 3D printers (Form 2 vs. Form 3). Results from three independent experiments are shown with a total of 12 biological replicates and the mean as a line. Statistical significance for each strain was assessed by a paired two-tailed Student’s t-test; a difference is considered significant at p < 0.05. ns, denotes not significant difference. **(C)** SEM images of silicone-coated peg (top) and dental resin-coated peg (bottom) at ×1,000 magnification (right, scale bars 20 µm) and ×150 magnification (left, scale bars 200 µm). **(D)** Viable cell count for 48-h biofilms grown on MBEC™/Calgary device or FlexiPeg with different types of pegs in Brain Heart Infusion (BHI) broth. **(E)** Representative pictures of biofilms grown on different pegs for 48 h and stained with 0.1% crystal violet (CV). **(F)** Amount of CV-stained biomass on different types of pegs quantified after dissolving the stain. Results for panels **(D, F)** are from two independent experiments with a total of eight biological replicates and the mean as a line. Results were compared by Brown–Forsythe and Welch ANOVA followed by Dunnett’s T3 multiple comparison test. p-Values only for significant differences (p < 0.05) are shown.

Having optimized the use of the FlexiPeg device with HT resin pegs, we further evaluated the effects of the additive manufacturing technology, different peg materials, and additional coatings. Since additive manufacturing works by the addition of material layer upon layer, 3D-printed pegs have a slightly rough surface (**Figure 3A**, middle) and thus increased surface area for attachment of bacteria. However, the roughness of the surface can be highly dependent on the specific additive manufacturing technology. We compared the same resin pegs printed on Form 2 (stereolithography technology) and a newer Form 3 (low force stereolithography technology) 3D printer. The SEM imaging confirmed that while the tip of the peg showed ridges of similar size (80–90 µm) (**Figure 3A**, right), the surface away from the tip, including the air–liquid interface, where most of the biofilm forms, had a much smoother surface on Form 3 HT pegs (**Figure 3A**, left). The material defects were also more prominent on Form 2 pegs (**Supplementary Figure 3**). While the strong biofilm-forming *K. pneumoniae* DA69557 did not show any difference, *K. pneumoniae* DA12090 had significantly lower CFU per peg on Form 3 printed pegs (**Figure 3B**), consistent with the smoother surface impeding the attachment of this strain.

We also printed pegs in dental SG resin, which is certified to be biocompatible (ISO 10993-1:2009 Biological Evaluation of Medical Devices) and is used in dental surgery. To achieve a smoother surface, we coated the pegs with the same resin after 3D printing (**Figure 3C**, top). In addition, we coated HT pegs with PDMS (silicone) to mimic the surface of catheters that are frequently made of silicone (Lawrence and Turner, 2005). These silicone-coated pegs showed the smoothest surface with the silicone completely filling in the ridges of the peg (**Figure 3C**, bottom). The peg material and coatings affected the growth of biofilms in a strain- and species-dependent manner (**Figures 3D** **–F**). The number of bacterial cells in 48-h biofilms grown on uncoated HT or dental resin pegs was at least 1–2 log higher than on polystyrene MBEC™ pegs for all strains (**Figure 3D**). Although CFU per peg was increased on custom-made pegs, the clear distinction between the two *K. pneumoniae* strains remained, proving that the results reflect differences in attachment and/or biofilm formation capabilities. The resin itself (HT vs. dental) did not affect the growth of *K. pneumoniae* strains, but *E. coli* had lower CFU numbers on dental resin pegs. The strongest biofilm-forming strain, *K. pneumoniae* DA69557, had consistently higher CFU per peg on all types of pegs as compared with MBEC™ and was not affected by the material or the tested coatings. The dental resin and silicone coating led to reduced CFU numbers for *K. pneumoniae* DA12090, while *E. coli* CFT073 was not affected by the silicone coating, indicating that it is not only the surface roughness but the material itself that is important to consider.

CV staining, which provides information on total biofilm biomass including ECM (Christensen et al., 1985; O’Toole, 2011), showed similar results as CFU counts (**Figures 3E, F**). CV staining requires a relatively high amount of biomass for detection (Stiefel et al., 2016), which made the quantification of *K. pneumoniae* DA12090 and *E. coli* CFT073 biofilms harder on certain pegs but made the difference to *K. pneumoniae* DA69557 even more prominent. As with CFU, *K. pneumoniae* DA12090 formed the most biofilm on uncoated pegs, while both dental resin and silicone coating reduced the biomass to the MBEC™ peg level. *E. coli* CFT073 had more biomass on HT pegs than on dental resin pegs, and silicone coating supported equally high biomass. *K. pneumoniae* DA69557 again performed better on all custom-made pegs compared with polystyrene MBEC™ pegs, with the HT pegs supporting the highest amounts of biomass. It is worth noting that the amount of biomass for this strain was so high that even prolonged de-staining did not completely dissolve the stain, although acetic acid (10%) was more efficient than ethanol (95%). However, this was not specific to our custom-made pegs, as we observed the same effect with MBEC™ pegs. The remaining stain was completely removed during washing of pegs in detergent for reuse of pegs. Silicone pegs have to be inspected more carefully over time, as they can retain the stain permanently when strong biofilm formers are grown. Also, a good indication motivating the replacement of silicone pegs is “hazy” as opposed to “glossy” appearance, or any peeling of the silicone layer. Compared with MBEC™, HT pegs absorbed more CV stain (absorbance at 540 nm HT 0.1731 ± SD 0.05329 vs. MBEC 0.042 ± SD 0.0008165, n = 4–8) increasing the detection threshold. The peg material and coatings led to different absorption of CV (**Supplementary Figure 4**), making the coated versions more suitable and easier to use for CV quantification.

**Figure 4:**
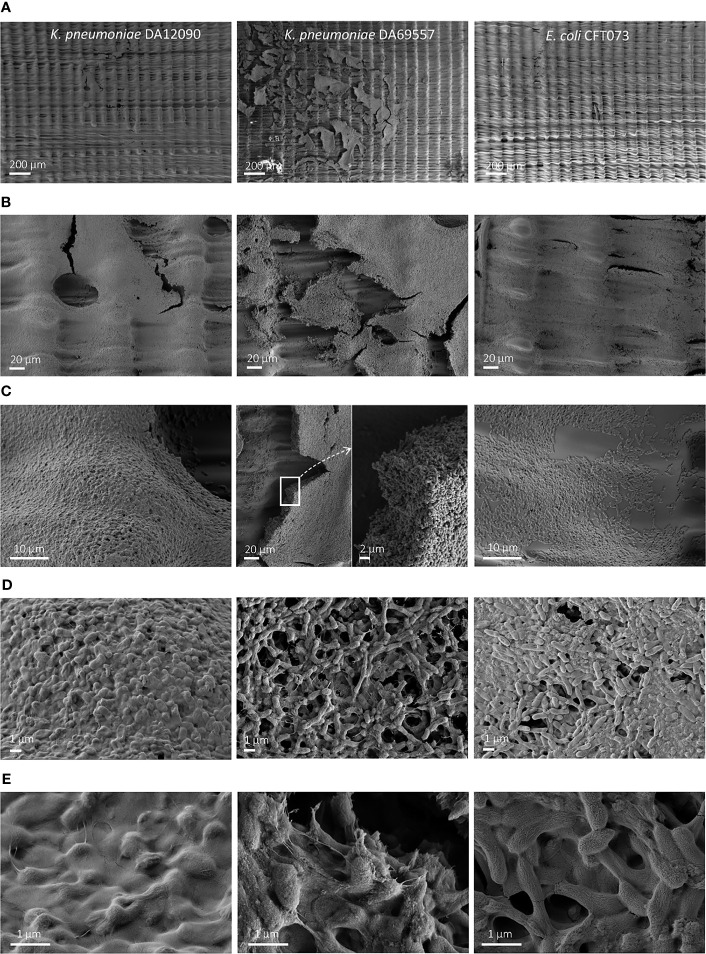
Scanning electron microscopy (SEM) imaging of 48-h biofilms on high-temperature resin (HT; Form 2 printer) pegs. *Klebsiella pneumoniae* DA12090 (left), *K. pneumoniae* DA69557 (middle), and *Escherichia coli* CFT073 (right) biofilms grown for 48 h in Brain Heart Infusion (BHI) medium and imaged at **(A)** ×150 magnification, **(B)** ×1,000 magnification, and **(C)** ×5,000 magnification for DA12090 and *E. coli*; ×1,000 with ×5,000 inset for DA69557, **(D)** ×15,000 magnification, and **(E)** ×50,000 magnification.

**Figure 5:**
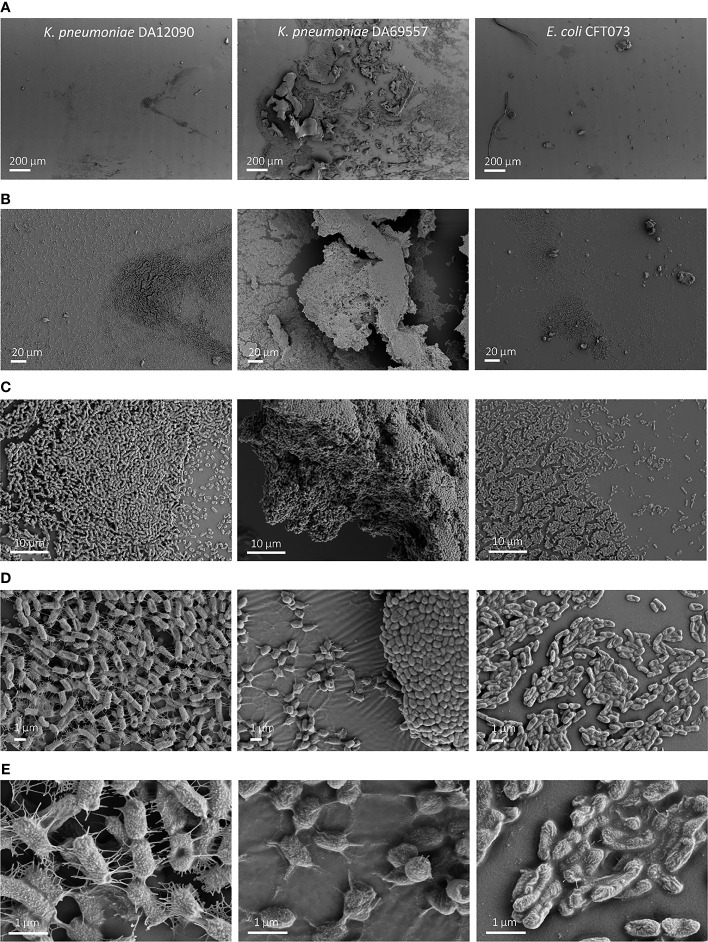
Scanning electron microscopy (SEM) imaging of 48-h biofilms on silicone-coated pegs. *Klebsiella pneumoniae* DA12090 (left), *K pneumoniae* DA69557 (middle), and *Escherichia coli* CFT073 (right) biofilms grown for 48 h in Brain Heart Infusion (BHI) medium and imaged at **(A)** ×150 magnification, **(B)** ×1,000 magnification, **(C)** ×5,000 magnification, **(D)** ×15,000 magnification, and **(E)** ×50,000 magnification.

To visualize the biofilm differences in more detail, we performed SEM imaging on biofilms grown for 48 h on uncoated HT resin pegs (**Figure 4**) and silicone-coated pegs (**Figure 5**). For all strains, we observed that most of the biofilm growth occurs at the air–liquid interface, approximately 4 mm from the tip of the peg. This was especially prominent for silicone-coated pegs. Consistent with CFU per peg and CV staining observations (**Figures 3D** **, F**), *K. pneumoniae* DA69557 produced massive amounts of biofilm with numerous layers of cells intertwined in an extensive ECM on both HT and silicone-coated pegs. In contrast, *K. pneumoniae* DA12090 and *E. coli* CFT073 showed a clear difference on HT versus silicone-coated pegs. DA12090 on HT pegs was embedded in a more continuous ECM and covered a larger area on the peg, while on silicone-coated pegs, the ECM looked more like a web of fibrils connecting the cells and the biofilm was thinner, confined to a smaller area on the peg, and there were more areas with single cells. Even though *E. coli* also showed quite a lot of ECM on HT pegs, most of the areas on these pegs had a monolayer appearance. We observed a patch-like monolayer distribution on silicone-coated pegs for *E. coli*, showing a clear difference between *K. pneumoniae* and *E. coli*. These results confirmed that both *K. pneumoniae* and *E. coli* could form mature biofilms characterized by the presence of cells and ECM, but there were clear morphological differences dependent on species, strain, and the surface on which the biofilms were formed.

In conclusion, our custom-made pegs are compatible with SEM imaging in addition to quantifying population size by CFU counts and staining biomass with CV. Also, the desired surface finish and material can be chosen based on experimental needs. Larger population sizes are supported by uncoated HT or dental pegs, while silicone- or dental resin-coated pegs give a higher resolution, allowing the detection of more subtle differences in biofilm formation capacity between different species or strains, especially if CV staining is performed.

### 3.3 Biofilm Growth Dynamics on High-Temperature and Silicone-Coated Pegs

**Figure 6:**
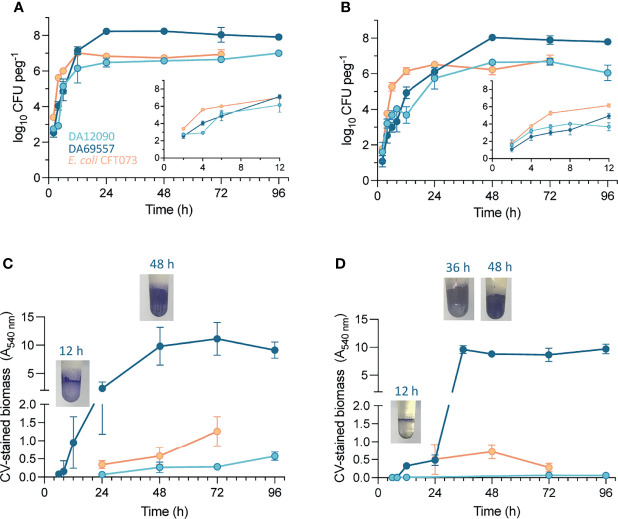
Change in biofilm population size and biomass on pegs over time. Viable cell count of *Klebsiella pneumoniae* DA12090 and DA69557 and *Escherichia coli* CFT073 populations from **(A)** high-temperature resin pegs and **(B)** silicone-coated pegs after vortexing. CFU counts were performed at different timepoints after peg inoculation with medium change every 24 h. The values are means of 3–6 biological replicates with standard deviations. Inset graphs in panels **(A, B)** represent magnification of the 2- to 12-h timepoints during biofilm growth. The biomass of *K pneumoniae* DA12090 and DA69557 and *E coli* CFT073 biofilms on **(C)** high-temperature resin pegs and **(D)** silicone-coated pegs stained with crystal violet. The images show the crystal violet (CV)-stained biomass of *K pneumoniae* DA69557 biofilms at respective timepoints.

To examine population size dynamics and biofilm maturation over time, we also assessed CFU per peg and quantified CV-stained biomass at different timepoints (2–96 h) on uncoated HT pegs (**Figures 6A, C**) and silicone-coated pegs (**Figures 6B, D**). On HT pegs, approximately 10^3^ CFU per peg were attached as early as 2 h after inoculation for all three strains (**Figure 6A**). When the medium was exchanged after 2 h to prevent the further attachment from the inoculum population, the growth curves maintained their initial trajectory (**Supplementary Figure 5**) showing that initiation of biofilm formation after 2 h is mostly driven by growth on the pegs and not by continuous attachment of planktonic cells from the liquid. *K. pneumoniae* biofilms kept growing until 24 h, after which there was no further increase in CFU to the end of the experiment at 96 h. The growth of *E. coli* CFT073 on the pegs was faster during early timepoints but also saturated earlier, at 12 h. An increase in CV-stained biomass (**Figure 6C**), indicating biofilm maturation, showed even bigger differences with *K. pneumoniae* DA69557 accumulating biomass as early as 12 h after inoculation, while the biomass gradually increased for the other strains. Bacterial attachment and growth on silicone-coated pegs (**Figure 6B**) were slower for both *K. pneumoniae* strains, while *E. coli* CFT073 behaved similarly as on HT pegs. CV-stained biomass dynamics also showed differences. For example, the ring at the air–liquid interface started showing already after 12 h for *K. pneumoniae* DA69557, but in contrast to HT pegs, maturation on the rest of the surface took longer before it sharply increased after 24 h. *K. pneumoniae* DA12090 barely gave a CV signal over the whole 96 h, and *E. coli* CFT073 showed a similar amount at 24 and 48 h but reached only half the final biofilm biomass at 72 h compared with HT pegs.

**Figure 7:**
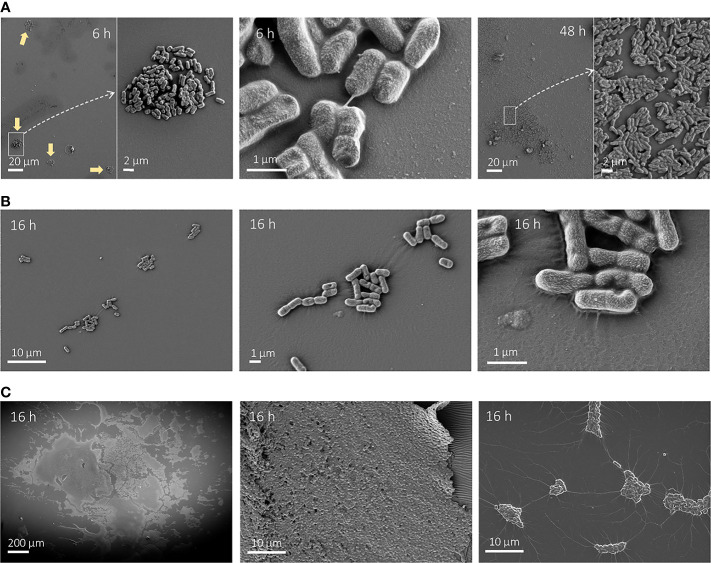
Scanning electron microscopy (SEM) images of growth on silicone-coated pegs at different timepoints. **(A)** *Escherichia coli* after 6 h (left and middle) or 48 h (right) growth imaged at ×1,000 magnification (left and right) and ×5,000 magnification. The typical cluster-like distribution at 6 h is denoted by yellow arrows. **(B)** *Klebsiella pneumoniae* DA12090 after 16 h of growth imaged at ×5,000 magnification (left), ×15,000 magnification (middle), and ×50,000 magnification (right). **(C)** *K. pneumoniae* DA69557 after 16 h of growth imaged at ×150 magnification (left) and ×5,000 (middle and right).

We also performed SEM imaging on HT and silicone-coated pegs at earlier timepoints to complement the 48-h observations described above. *E. coli* CFT073 showed small clusters of approximately 100 cells scattered on silicone-coated pegs as early as 6 h after inoculation (**Figure 7A**, left). The clusters contained dividing cells illustrating active growth on the peg, and some showed thin ECM fibers (**Figure 7A**, middle). In contrast, we only saw single cells sparsely attached to the HT peg for both *K. pneumoniae* strains (**Supplementary Figure 6**), which is in accordance with lower CFU per peg counts on silicone-coated pegs at 6 h compared with *E. coli* CFT073. It is worth noting that these single cells showed signs of excreted ECM covering the surface of the peg. For *K. pneumoniae* strains, we also chose a later timepoint at 16 h (**Figures 7B, C**), which clearly illustrated the difference in biofilm formation dynamics between these two strains. While DA12090 showed only a few cell clusters (**Figure 7B**, left and middle), a web-like ECM was protruding from the cells and covering the peg surface (**Figure 7B**, right). DA69557 displayed abundant biofilm tightly packed together already at this timepoint (**Figure 7C**). In addition, we noticed unique “connections” between different cell clusters in the areas of lower cell coverage for this strain (**Figure 7C**, right).

Overall, these dynamic observations provided more information than the end-point comparisons and revealed different biofilm growth and maturation rates dependent on surface, species, and strain.

## 4 Discussion

The concept of the FlexiPeg biofilm device was inspired by the CBD/MBEC™ device with the aim of keeping its high-throughput characteristics while also simplifying the handling of pegs, reducing the cost of material, and making the system more versatile in terms of applicability to different experimental setups. We achieved this by designing a device in which individual pegs are inserted into a lid, rather than being permanently attached. The manual and individual insertion of the pegs means that their numbers and locations can be easily adapted to specific experimental needs. In addition, because the pegs can simply be pushed from the top for removal, there is no need to come close to the biofilm growth surface itself, in contrast to the manipulation required for working with CBD (Ceri et al., 1999; Harrison et al., 2010) or the bead assay (Konrat et al., 2016). This design minimizes the risks of contamination or unwanted disturbance of the biofilms. In general, the design makes it easier to handle pegs for biofilm disruption, imaging, and any other desired end-point analysis of the biofilm population. We showed that this alternative design of the peg lid does not compromise the robustness of the assay and enables the growth of multiple equivalent biofilms that can be harvested at required timepoints for further analysis or exposure to additional treatments. The whole model system is easily manufactured by 3D printing, is reusable after sterilization, and does not require any specialized equipment to assemble or operate. Even though the work presented here is based on a 24-peg lid version fitting in a 96-well plate, which we find more convenient to work with than a 96-peg lid, the concept is easily applicable to changes in the arrangement to fit microtiter plates or trays of different sizes or to produce bigger pegs.

The type of surface material affects the growth of biofilms (Slettengren et al., 2020). Thus, the possibility for the user to choose peg material extends the versatility of our biofilm device even further, and pegs made out of different additive manufacturing materials or with certain coatings can be accommodated on the same lid. Modern 3D printing facilities can accommodate many different materials and post-printing surface modifications including stainless steel, aluminum, titanium, gold, and a wide range of composite materials. The pegs can also serve as a scaffold for coatings with other materials; for example, we coated pegs with silicone after 3D printing. The possibility of having multiple different materials is also provided by the CDC biofilm reactor (Goeres et al., 2005) that can hold 24 coupons exposed to the movement of the medium, and thus, generate high shear forces. However, it is not suitable for high-throughput analysis, as it only accommodates one strain per run and requires high amounts of growth medium.

In this proof-of-concept work, we examined *K. pneumoniae* and *E. coli* biofilm growth on two different resins (HT and dental) and applied additional coatings of either dental resin or silicone (PDMS), mimicking the surfaces of medical devices. Although unwanted in an *in vivo* situation with implants (Palka et al., 2020), here, we took advantage of the characteristic surface roughness typical of additive manufacturing. We showed that uncoated 3D-printed pegs with higher surface roughness supported larger population sizes (CFU per peg) and biomass (CV staining) than polystyrene MBEC™ pegs, making them suitable for fundamental biofilm studies requiring more biomass. We could not find any inconsistencies between biofilm formation on different individual pegs (printed with the same technology in different batches) showing that whatever differences may exist in the structure between different batches of pegs does not affect the outcome of the assay. However, surface features, such as roughness or hydrophobicity, determined by the peg material itself, the additive manufacturing technology, or different coatings, led to differences in biofilm formation in a species- or strain-dependent manner. This is in agreement with a recent study (Hall et al., 2021) that suggested that antibacterial properties (e.g., incorporated metals), surface roughness, and hydrophobicity are the major determinants for biofilm growth by *E. coli*, *P. aeruginosa*, and *Staphylococcus aureus* on 3D-printed polylactic acid-based materials. The authors observed less biofilm growth on polymers filled with metals as well as on smoother and less hydrophobic surfaces; however, the strongest biofilm-forming strain in their study (*P. aeruginosa*) did not show the same correlation. Similarly, while the weaker biofilm formers in our study, *K. pneumoniae* DA12090 and *E. coli* CFT073, showed growth and overall biomass differences dependent on the surface roughness or other material-dependent features, *K. pneumoniae* DA69557 grew equally massive biofilms on all materials and coatings tested. This shows how important it is to include several species or strains with varying biofilm features when testing different materials or choosing the right material when studying specific biofilms.

All three clinical strains tested were able to grow and form biofilms on our custom pegs, although with differences in the absolute amounts. All biofilms showed clear increases in CFU per peg, dividing cells during SEM imaging, and signs of maturation over time with the production of ECM and formation of other structures, such as water channels, visible in the SEM images. Moreover, the differences between the species and strains were clear and consistent in all assays. While the absolute values of CFU per peg or CV staining are difficult to compare when biofilms are grown in different models, SEM imaging allows direct visualization of a biofilm. We do acknowledge that sample preparation, including fixation and dehydration, can affect the biofilm, which is usually highly hydrated (Flemming and Wingender, 2010). However, all of the biofilms have undergone growth and sample preparation under the same conditions, and the SEM observations corresponded well with CFU and CV staining data. Therefore, we believe the differences observed are attributable to the actual differences in the biofilm morphology of these strains.

One important question is how relevant these biofilm observations are, especially given that the FlexiPeg device is a static batch-type model. Shear forces affect the strength of attachment and the structure of a biofilm (Stoodley et al., 2002) and consequently other features, such as susceptibility to antibiotics (Buckingham-Meyer et al., 2007). On the other hand, the extent of shear forces that biofilms are exposed to can vary greatly depending on the environment, making models with the high fluid flow not necessarily the most representative. The patchy distribution of CAUTI-associated *E. coli* SM1 on silicone catheters and the more “messy” three-dimensional appearance of *K. pneumoniae* SDM3 were reported after 72-h growth in artificial urine under flow conditions (Macleod and Stickler, 2007), which is in line with our observations of *E. coli* versus *K. pneumoniae* biofilms on silicone-coated pegs. These observations seem to extend to other studies of biofilms growing with or without flow. Wilks et al. also showed clustering of *E. coli* NCTC 9001 (UTI isolate), grown for 6 h on silicone catheters incubated in artificial urine (Wilks et al., 2021), which is in accordance with our observations at 6 h, even though the appearance at later timepoints differ. Both clusters and patchy monolayers were observed in different locations of silicone catheters, as a result of *E. coli* WT F1693 (CAUTI-associated) migrating along the catheter surface 7 days post-inoculation (Zhang et al., 2019). Different locations of silicon/latex catheters in a flow-based modified Robin’s device also had different stages of *E. coli* CFT073 growth with clusters, patchy distribution, and multilayer biofilms (Koseoglu et al., 2006). In addition to biofilms grown under laboratory conditions, ECM fibers were visible in *E. coli* biofilms on urinary catheters taken from patients (Wang et al., 2009).

Comparisons with previous studies for *K. pneumoniae* biofilms are more difficult, as the biofilms of *K. pneumoniae* are highly dependent on the strain (Bandeira et al., 2014; Bandeira et al., 2017), and in general, there are less published SEM data on *K. pneumoniae* biofilms. Nevertheless, fibrils of ECM connecting the cells in mature biofilms have been shown for *K. pneumoniae* (Balestrino et al., 2008). Such structures were also observed for *K. pneumoniae* grown in a mixed biofilm with *Candida albicans* on a urinary catheter isolated from a patient (Vuotto et al., 2014). Abundant *E. coli* common pilus (ECP) filaments connecting *K. pneumoniae* aggregating on HeLa cells (Alcántar-Curiel et al., 2013) are reminiscent of our observations for *K. pneumoniae* DA69557 at 16 h with clear connections between the clusters of cells on silicone-coated pegs. Therefore, although in-depth functional analysis of biofilms is outside the scope of this study, data obtained using the FlexiPeg device point to similarities with other models, including those with flow, and actual clinical observations.

The major limitations of our biofilm device are linked to the inability to sample or image live biofilm during growth. While CBD pegs have been imaged with confocal laser scanning microscopy (CLSM) (Harrison et al., 2006), it was still an endpoint analysis rather than a live *in situ* imaging that is possible with, e.g., flow-cell based systems. CLSM imaging allows for the observation of the matrix in its fully hydrated form or for marking certain components, which is not possible with SEM, even though SEM offers a much higher resolution. Also, it is not possible to sample the biofilm during growth, and time dynamics-based experiments rely on biofilms being formed equally on all pegs to make comparisons possible. While we have shown that biofilms formed at 48 h indeed do not show any difference due to peg location, the biological variation (e.g., in terms of time to biofilm maturation) between replicates at earlier timepoints or when applying certain treatments can make the analysis more challenging compared with a situation where the same biofilm is analyzed before and after treatment, or at different timepoints. Although the validation of the system presented here was done on Enterobacteriaceae strains, we do not see any constraints in studying other microbes, as many have been shown to form biofilms on 3D-printed materials (Palka et al., 2020; Hall et al., 2021), and silicone devices (Singhai et al., 2012).

In conclusion, the FlexiPeg biofilm device represents a simple, low cost, and relevant assay to study biofilms on diverse surfaces, including but not limited to additive manufacturing materials. The modular nature of the assay enables diverse experimental setups and the option of up- or down-scaling, making it an attractive choice for screening purposes, susceptibility testing, and general biofilm studies.

## Data Availability Statement

The original contributions presented in the study are included in the article/**Supplementary Material**. CAD-files for the 3D-printing of the FlexiPeg device can be downloaded here: https://www.imbim.uu.se/digitalAssets/675/c_675902-l_1-k_flexipeg_cad_drawings.zip. Further inquiries can be directed to the corresponding author.

## Author Contributions

GZ: method validation, experiments, data analysis, writing—original draft, review, and editing. EW-Y: device design and development, and writing—review and editing. KH: experiments, data analysis, and writing—review and editing. DA: supervision and resources, and writing—review and editing. LS: supervision and resources, and writing—review and editing. All authors read and approved the final version of the manuscript.

## Funding

Funding was provided by Carl Tryggers stiftelse (CTS 19:315) to LS and by the Swedish Research Council (Vetenskapsrådet) (2017-01527) and the Thon Foundation to DA. MyFab national research infrastructure receives partial support from Swedish Research Council and the Wallenberg Foundations.

## Conflict of Interest

EW-Y is currently employed by Astrego Diagnostics AB. The company had no part in the design, performance, or analysis of data in the study.

The remaining authors declare that the research was conducted in the absence of any commercial or financial relationships that could be construed as a potential conflict of interest.

## Publisher’s Note

All claims expressed in this article are solely those of the authors and do not necessarily represent those of their affiliated organizations, or those of the publisher, the editors and the reviewers. Any product that may be evaluated in this article, or claim that may be made by its manufacturer, is not guaranteed or endorsed by the publisher.
