# Supplementary material for: Modular 3D-Printed Peg Biofilm Device for Flexible Setup of Surface-Related Biofilm Studies
Source: Front Cell Infect Microbiol. 2022 Feb 3;11:802303. doi: 10.3389/fcimb.2021.802303 (PMC8851424; doi:10.3389/fcimb.2021.802303)
Supplement: Supplementary file 1 [file DataSheet_1.pdf]

Supplementary material

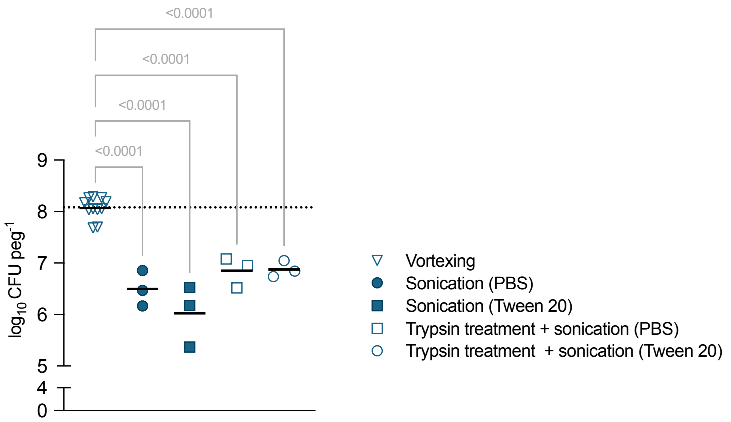

Supplementary Figure 1. The effect of different methods for biofilm disruption. Biofilms were formed on HT pegs for 48 h in BHI medium by DA69557 strain. Sonication in an ultrasonic bath was carried out for 10 min. Dotted line represents the mean value obtained when vortexing the biofilm for 2 min full speed

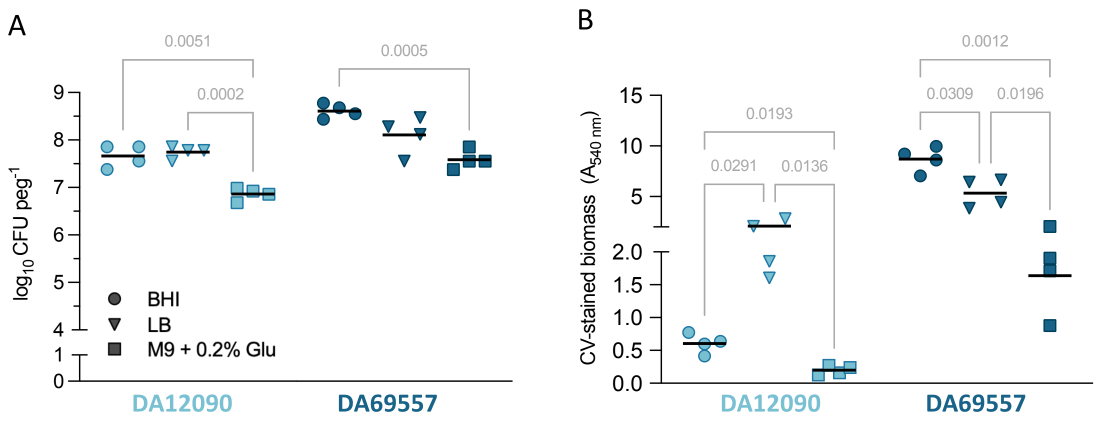

Supplementary Figure 2. *K. pneumoniae* biofilm growth (48 h) on HT pegs in different media determined by (**A**) CFU per peg and (**B**) CV-stained biofilm biomass. Results show four biological replicates and the line represents the mean. Statistical significance assessed by Brown-Forsythe and Welch ANOVA followed by Dunnett's T3 multiple comparison test. P values only for significant differences ($P < 0.05$) are shown.

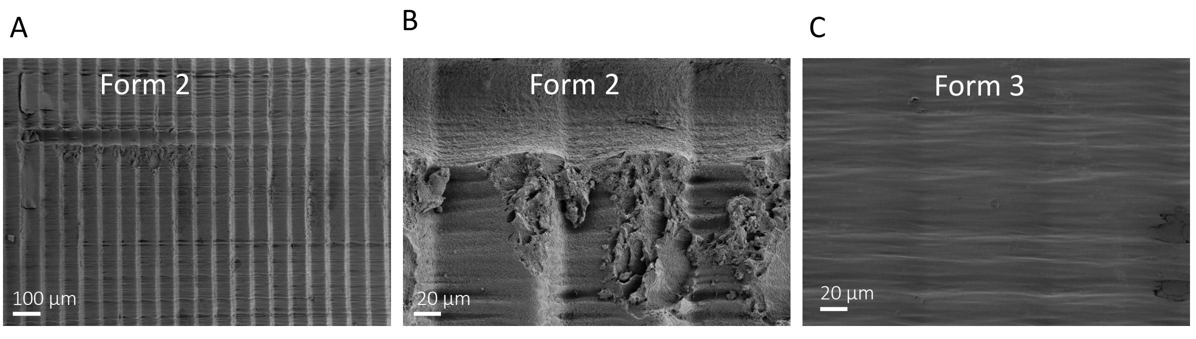

Supplementary Figure 3. SEM images of HT pegs printed on Form 2 printer (**A**, 150x magnification and **B**, 1000x magnification) and Form 3 printer (**C**, 1000x magnification).

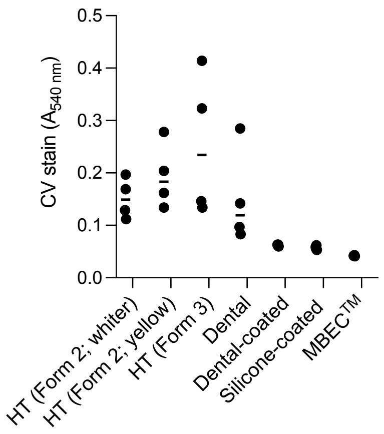

Supplementary Figure 4. CV stain absorption on control pegs (incubated in BHI) without any biofilm.

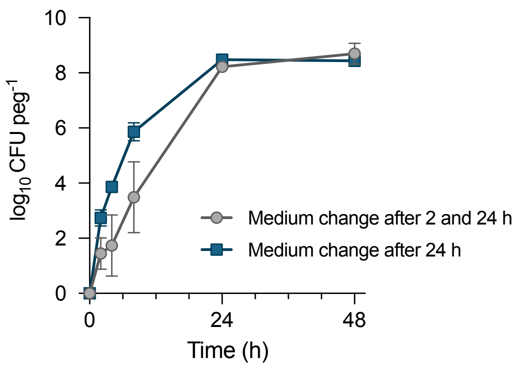

Supplementary Figure 5. Growth curve of *K. pneumoniae* DA69557 on HT pegs with a medium change at different timepoints. Results show mean of four biological replicates with standard deviations.

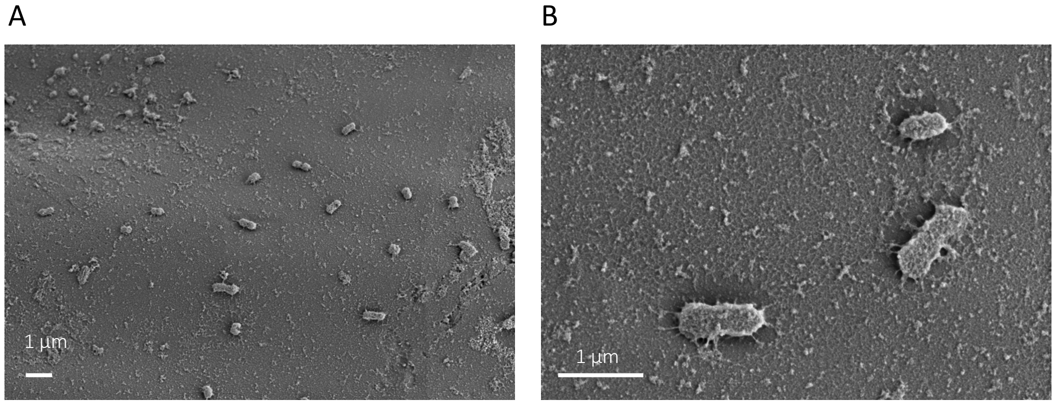

Supplementary Figure 6. SEM images of *K. pneumoniae* DA69557 on HT pegs 6 h after inoculation. (**A**) 15000x magnification and (**B**) 50000x magnification.
